# Mass Spectrometry-Based Peptidomics for the Discovery and Profiling of Endogenous Peptides in Crustacean Hemolymph

**DOI:** 10.1021/acsomega.6c00679

**Published:** 2026-05-05

**Authors:** Tina C. Dang, Vu Ngoc Huong Tran, Satirtha Saha Protya, Margot Beaver, King Wai Yi, Lingjun Li

**Affiliations:** † School of Pharmacy, 5228 University of Wisconsin-Madison, Madison, Wisconsin 53705, United States; ‡ Madison West High School, 30 Ash Street, Madison, Wisconsin 53726, United States; § Department of Chemistry, University of Wisconsin–Madison, Madison, Wisconsin 53706, United States; ∥ Lachman Institute for Pharmaceutical Development, School of Pharmacy, University of Wisconsin–Madison, Madison, Wisconsin 53705, United States; ⊥ Wisconsin Center for NanoBioSystems, School of Pharmacy, University of Wisconsin–Madison, Madison, Wisconsin 53705, United States

## Abstract

Crustacean hemolymph contains a diverse array of bioactive peptides, including antimicrobial peptides (AMPs) and neuropeptides, which regulate essential immune and physiological processes. However, rapid coagulation and the presence of high-abundance proteins complicate mass spectrometry (MS)–based peptidomic analysis. To address these issues, we systematically evaluated five extraction workflows to optimize endogenous peptide recovery from the hemolymph of the American lobster (*Homarus americanus*) and the blue crab (*Callinectes sapidus*). Among the tested approaches, Method 3, which combines anticoagulants and protease inhibitors with a 10 kDa molecular-weight-cutoff filtration step, achieved the highest peptide identification rates. This workflow minimized clotting-related peptide loss and enzymatic degradation, resulting in markedly improved recovery of AMPs with diverse hydrophobicity profiles in both species. Notably, omitting the reduction and alkylation steps in Method 3 further enhanced AMP recovery. For example, in *C. sapidus*, inclusion of these steps (Method 4) led to an approximately 40% decrease in AMP identifications, likely due to increased sample handling and prolonged incubation. Method 3 also maximized neuropeptide recovery in *H. americanus*, enabling identification of predicted mature orcokinins and myosuppressins. In *C. sapidus*, neuropeptide recovery varied across workflows, with Method 3 providing competitive performance. Additionally, we report six novel putative neuropeptides in *C. sapidus* and identify nine and 11 *in silico*-predicted novel AMPs in *C. sapidus* and *H. americanus*, respectively, characterized by distinct amino acid motifs. Collectively, this study establishes an integrated extraction strategy that enables more comprehensive profiling of crustacean hemolymph peptidomes and provides the first combined report of predicted AMPs and neuropeptides in *C. sapidus* and *H. americanus*. These findings expand the repertoire of candidate molecules with potential roles in antimicrobial defense and neuromodulation.

## Graphical abstract

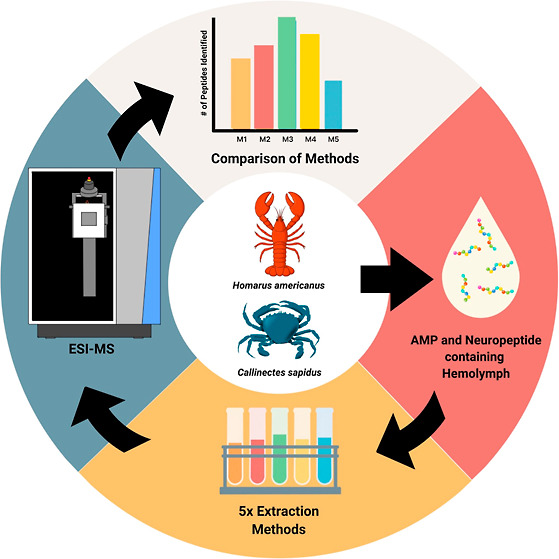

## Introduction

Endogenous peptides, including antimicrobial peptides (AMPs) and neuropeptides, play essential roles in immunity, neural signaling, and physiological homeostasis.^1^ AMPs are key components of the innate immune system across all domains of life and are often referred to as “natural antibiotics” due to their broad-spectrum activity against bacteria, fungi, parasites, and viruses.^2,3^ With the rapid rise of bacterial resistance to conventional antibiotics, AMPs have gained increasing attention as promising candidates for next-generation antimicrobial therapeutics.^4^ Neuropeptides constitute a structurally diverse and heterogeneous class of signaling molecules. Functionally, they are highly diverse, acting as neurotransmitters, neuromodulators, or hormones that regulate or coregulate activity across the nervous and endocrine systems.^5^ After being released from neuroendocrine cells, many neuropeptides enter the circulating fluid, where they act at distant targets to regulate key physiological processes such as feeding behavior, stress response, and nociception, making them essential mediators of neural-endocrine communication.^6–10^ In this work, we use the term “neuropeptides” broadly to encompass both neuronal and hormonal signaling peptides, in alignment with established neuropeptide databases. Despite their biological significance, endogenous peptides such as AMPs and neuropeptides are challenging to profile due to their low abundance, susceptibility to degradation, and diverse post-translational modifications (PTMs), requiring highly sensitive analytical methods such as mass spectrometry (MS) for reliable characterization.

Crustaceans provide a unique and highly advantageous model for the discovery and characterization of both AMPs and neuropeptides. Like other invertebrates, crustaceans lack adaptive immune components such as immunoglobulins, lymphocytes, and memory responses, relying exclusively on the innate immunity system, which consists of cellular and humoral responses as a defense mechanism.^3^ The cellular response is mediated primarily by hemocytes circulating in the hemolymph, while the humoral response is driven largely by AMPs. Because these AMPs are produced predominantly by hemocytes, the hemolymph serves as a rich and chemically diverse reservoir of these immune-active peptides.^11^ In parallel, crustacean hemolymph provides direct and convenient access to diverse neuropeptides secreted from neuroendocrine organs, many of which share homology with mammalian counterparts and thus offer broad relevance for comparative biological research.^12^ Species such as the American lobster (*Homarus americanus*) and the blue crab (*Callinectes sapidus*) are not only biologically advantageous models for the discovery of novel AMPs from natural resources but have also served as long-standing models in neuropeptide research, underscoring the importance of characterizing their immune and neuropeptide profiles in the hemolymph.

Although hemolymph is an ideal biological matrix for AMPs and neuropeptide discovery in crustaceans, its complex properties pose substantial challenges for peptidomic analysis. Upon hemolymph withdrawal, the hyaline hemocytes rapidly aggregate and initiate the coagulation cascade, which can entrap circulating endogenous peptides and thereby reduce extraction efficiency.^13–15^ Additionally, endogenous proteases present in hemolymph further jeopardize full-length peptide integrity, complicating accurate characterization. To mitigate these issues, several strategies have been utilized, including the use of acidified organic-solvent precipitation, anticoagulants, and protease inhibitors to preserve native peptide profiles throughout extraction and analysis.^16–18^ Furthermore, many crustacean AMPs and neuropeptides are cysteine-rich and stabilized by multiple intramolecular disulfide bonds.^3,19^ These structural features often require additional experimental procedures (e.g., reduction and alkylation) to fully resolve disulfide linkages for MS detection.^20,21^ Collectively, the instability of hemolymph, its high proteolytic activity, and the complexity of disulfide-rich peptides underscore the need for a carefully optimized extraction protocol to maximize recovery of endogenous AMPs and neuropeptides from this complex biological matrix.

MS-based peptidomics offers significant advantages for the characterization of endogenous peptides over traditional biochemical assays, enabling high sensitivity, PTMs detection, and isoform differentiation.^22,23^ However, the success of any MS-based experiment depends critically on sample preparation and endogenous peptide extraction. Buffer composition, the inclusion of anticoagulants or protease inhibitors, and reduction/alkylation conditions can each bias the subset of peptides detected, thereby affecting the discovery of both known and novel AMPs and neuropeptides. To disentangle these effects, a systematic comparison of peptide extraction methods optimized specifically for crustacean hemolymph is urgently needed. It should be noted that, due to the broad discovery capability of MS-based peptidomics, the peptides detected in the hemolymph may include previously characterized AMPs and neuropeptides as well as newly observed peptide candidates annotated based on sequence homology and precursor information, without necessarily confirming their cellular origin, receptor interactions, or biological activity. Within this work, the terms “AMPs” and “neuropeptides” serve as shorthand for the respective detected peptides in the data set; however, we emphasize that these designations remain sequence annotation-based, with novel sequences explicitly labeled as “putative” to reflect their status as newly observed candidates requiring future functional validation.

In this study, we developed an optimized MS-based workflow tailored to recover endogenous, putative AMPs and neuropeptides from the hemolymph of the American lobster and blue crab. We conducted a side-by-side evaluation of five extraction protocols incorporating combinations of anticoagulants, protease inhibitors, and cysteine-resolving chemistries. Through systematic assessment, we identified key methodological biases and established an optimized extraction strategy that improves intact peptide preservation and detection. This streamlined workflow enhances AMP and neuropeptide coverage and generates high-quality data sets for comparative and functional studies of crustacean immune and neuroendocrine signaling.

## Materials and Methods

### Chemicals and Materials

Unless noted otherwise, all chemicals and solvents were purchased from Fisher Scientific (Pittsburgh, PA, USA). ACS-grade formic acid (FA) was purchased from Sigma-Aldrich (St. Louis, MO, USA). Amicon Ultra 10 kDa 0.5 mL molecular weight cutoff (MWCO) devices, 10 μL C18 ZipTips, and Roche cOmplete, Mini, EDTA-free protease inhibitor cocktail were purchased from MilliporeSigma (Burlington, MA, USA). 100 μL Omix tips were obtained from Agilent (Santa Clara, CA).

### Animals

American lobsters and blue crabs were purchased from Global Market and Food Hall (Madison, WI) and acclimated to the tank (12 °C, 12 h dark/light cycle) for at least 3 weeks prior to any experiment. Crustaceans were anesthetized on ice for 30 min before hemolymph withdrawal. A 25-gauge needle attached to a 1 mL BD plastic syringe was inserted through the first junction of the thorax and abdomen into the pericardial chamber to withdraw the hemolymph. A total of 5 mL of hemolymph was withdrawn from each animal. One milliliter of hemolymph was allocated to each of the five extraction workflows (Figure 1). Three biological replicates were analyzed for each species.

**Figure 1:**
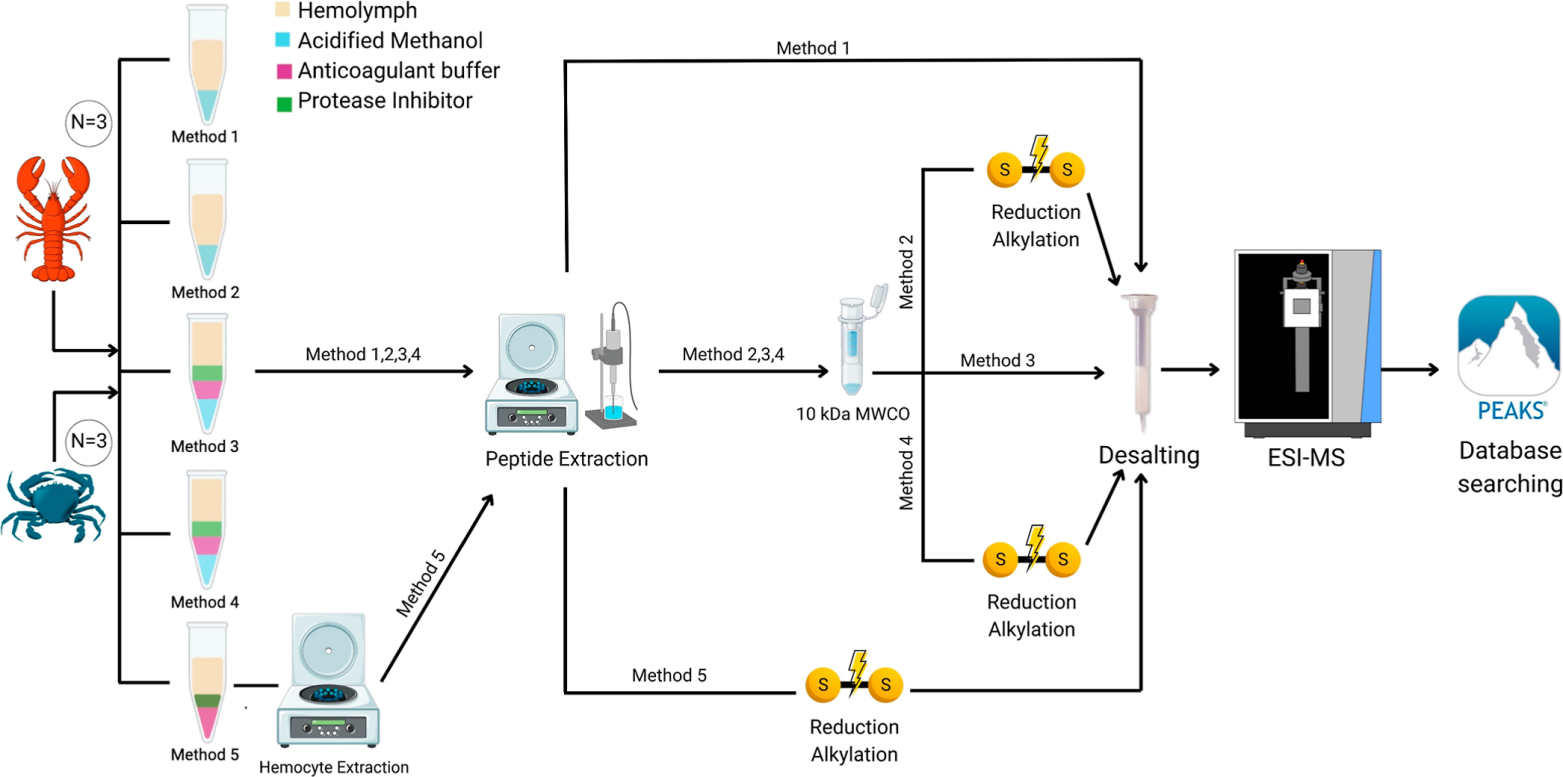
Schematic of the five workflows evaluated for hemolymph collection and endogenous peptide extraction from American lobsters and blue crabs for in-depth mass-spectrometry-based peptidomics, differing in the use of anticoagulants, protease inhibitors, and reduction/alkylation reagents.

### Endogenous Peptide Extraction from Hemolymph

#### Method 1

One mL of hemolymph was added to 1 mL of chilled acidified methanol (AcMeOH 90/9/1 v/v/v methanol/water/glacial acetic acid), vortexed, incubated on ice for 20 min, and centrifuged at 14,000 rcf at 4 °C for 10 min. The supernatant was collected and transferred to a Protein Lo-Bind 2 mL tube. 0.5 mL of AcMeOH was then added to the pellet, and an electric sonicator was used to break the precipitate and produce a homogenized solution in an ice bath. The sonicator was set to 15 s on and 15 s off for 1 min at 60% amplitude for all samples. Samples were centrifuged at 14,000 rcf for 10 min, and the supernatant was collected and combined with the previous supernatant. This process was repeated one more time for a total of three extractions. The supernatant was dried down in a vacuum centrifuge (SpeedVac) on medium heat and stored at −80 °C until needed.

#### Method 2

The protocol for method 1 was followed exactly until after the last cycle of probe sonication and centrifugation. The sample was then subjected to a 10 kDa MWCO filter. The filter was first rinsed with 0.2 mL of 0.1 M NaOH in the centrifuge at 14,000 rcf for 4 min, and then rinsed again with 0.5 mL of AcMeOH for 8 min at 14,000 rcf. The sample was added to the filter and centrifuged at 16,400 rcf until most of the liquid had passed through. Once complete, the filter was rinsed once more with 0.1 mL of AcMeOH for 20 min at 14,000 rcf, and the flow-through was collected.

Next, Dithiothreitol (DTT) was added to a final concentration of 5 mM and incubated for 30 min at 37 °C. After DTT treatment, Iodoacetamide (IAA) was added to a final concentration of 10 mM and incubated for 30 min in the dark at room temperature. The same volume of DTT was added to the sample to quench the alkylation, and the supernatant was dried down on medium heat and stored at −80 °C until needed.

#### Methods 3 and 4

Five mL of 2×-concentrated anticoagulant buffer (AB) (0.9 M NaCl, 0.2 M glucose, 60 mM trisodium citrate, 52 mM citric acid and 20 mM EDTA, pH = 4.6); and one tablet of protease inhibitor (PI) dissolved in 40 mL of 20 mM EDTA were prepared. For both methods 3 and 4, 0.5 mL of AB and 0.5 mL of PI were combined with 1 mL of hemolymph, vortexed well, and incubated on ice for 20 min. Two mL of AcMeOH were then added, vortexed, ice-bath sonicated for 5 min, and centrifuged at 16,100 rcf at 4 °C for 10 min. The supernatant was collected, and a total of three extractions were performed as outlined above. The samples were subjected to a 10 kDa MWCO device. The flow-through for method 3 was dried down in the speedvac on medium heat and stored at −80 °C until needed. The flow-through for method 4 was treated with DTT and IAA as described in method 2 before being dried down and stored at −80 °C.

#### Method 5

0.5 mL of AB and 0.5 mL of PI were combined with 1 mL hemolymph, vortexed, incubated on ice for 20 min, and centrifuged at 800 rcf for 10 min. The supernatant was discarded, and the hemocyte pellet was retained. One mL of AcMeOH was added to the pellet, probe sonicated, and centrifuged again at 14,000 rcf for 10 min. The supernatant was transferred to a clean tube and treated with DTT/IAA as outlined in method 2. The sample was dried down in the SpeedVac on medium heat and stored at −80 °C.

All samples were desalted first with Omix tips, and second with C18 Ziptips in 2 elution steps (E1 50% ACN in 50% H2O with 0.1% FA and E2 75% ACN in 25% H2O with 0.1% FA) following the manufacturer’s protocol prior to mass spectrometry injection. The eluate was dried down in the SpeedVac on medium heat and stored at −80 °C until mass spectrometer analysis.

### LC–MS/MS Acquisition

Before injection into the mass spectrometer, the samples were resuspended in 20 μL of 0.1% FA, and their concentrations were measured using a Thermo Scientific NanoDrop one microvolume spectrophotometer to ensure around 500 ng of sample per injection. Samples were analyzed on the Thermo Orbitrap Exploris 480 mass spectrometer coupled with a Vanquish Neo UHPLC system. A self-packed Waters BEH C18 particles 19 cm microcapillary column was used at a 0.3 μL/min flow rate with mobile phase A (0.1% FA), and mobile phase B (0.1% FA in 80% acetonitrile).

*C. sapidus* samples were run on a 126 min chromatographic separation as follows: 0–60 min 3–40% B; 60–90 min 40–70% B; 90–90.5 min 70–90% B; 90.5–105.5 min 90% B; 105.5–106 min 90–100% B; 106–126 min 100% B. MS scan range was 200 to 2000 *m*/*z* at a resolution of 75,000, RF Lens of 30%, a normalized AGC target of 100%, and maximum injection time of 250 ms. Precursors were subject to dynamic exclusion for 35 s with a 10 ppm tolerance, and the top 20 most intense precursors were subsequently selected for MS2 fragmentation with a normalized collision energy of 30%, a resolution of 15,000, a standard AGC target, and a maximum injection time of 150 ms.

*H. americanus* samples were run on a 90 min chromatographic separation as follows: 0–7 min 3–15% B; 7–37 min 15–27.5% B; 37–52 min 27.5–40% B; 52–72 min 40–70% B; 72–77 min 70–100% B; 77–90 min 100% B. MS scan range was 200 to 1500 *m*/*z* at a resolution of 60,000, RF Lens of 50%, a normalized AGC target of 300%, and auto maximum injection time. Precursors were subject to dynamic exclusion for 30 s with a 10 ppm tolerance and selected for MS/MS using a 3 s top-speed data-dependent acquisition cycle. MS2 spectra were acquired with a normalized collision energy of 30%, a resolution of 15,000, a standard AGC target, and a maximum injection time of 40 ms.

### Database Searching

All MS raw data were searched against various databases using PEAKS Studio XPro (Bioinformatics Solutions Inc.). For the *C. sapidus* data, two specific databases were utilized, the crustacean neuropeptide (cNPDB)^24^ and the AMPSphere database,^25^ which contains sequences predicted *in silico* from crustacean metagenomic data. For *H. americanus* data, the *H. americanus* proteome database, containing 24,734 protein entries, was downloaded from UniProt on Feb 12th, 2023. Specialized databases comprising known AMPs^3^ and neuropeptide^12,19^ precursors were further curated meticulously based on protein annotations. The search parameters include 10 ppm parent mass error tolerance, 0.02 Da fragment mass error tolerance, and an unspecific digest mode. A maximum of 3 variable PTMs, including C-terminal amidation, oxidation (M), N-terminal pyro-gln (Q), and N-terminal pyro-glu (E), were allowed. The identified peptides were filtered using the following thresholds: Proteins −10lgP ≥ 0, ≥ 1 unique peptide, and with significant peptides; Peptides −10lgP ≥ 20.^26^ Peptides detected in at least two of the three biological replicates were considered positively identified. Subsequent data processing and visualization were further conducted using Microsoft Excel and custom Python scripts. Putative neuropeptides detected in *C. sapidus* hemolymph were first obtained through de novo sequencing results from PEAKS XPro and retained only if they met a Total Local Confidence (TLC) threshold of at least 85%. These candidate sequences were then searched against known crustacean neuropeptide motifs^12,19^ using a custom Python script. To verify each assignment, MS/MS spectra were manually inspected to ensure fragment ions supported cleavage across the peptide backbone and confirmed the proposed sequence.

### AMPs Prediction

For the prediction of potentially novel AMP candidates, we first utilized PEAKS Studio XPro to generate de novo peptide sequences, filtering the results stringently at a Total Local Confidence (TLC) of 85%. These sequences were then formatted into a FASTA file and analyzed using MotifQuest.^27^ MotifQuest was used to identify and parse out biologically relevant amino acid motifs that were conserved across multiple peptide sequences. The resulting motif containing peptide sequences were then cross-referenced against established AMPs databases^25,28^ to ensure their novelty. Sequences confirmed as novel were assembled for *in silico* validation using three independent prediction tools: DBAASP (Database of Antimicrobial Peptides and Structures),^29^ AMPScanner,^30^ and CAMPR3′s artificial neural network classifier model.^31^ A sequence was reported as a putative novel AMPs (Table S1) only if DBAASP, AMPScanner, and CAMPR3 independently agreed upon its antimicrobial potential.

## Results and Discussion

### Antimicrobial Peptides

Five different extraction protocols (Methods 1–5) were compared to assess their efficiency in isolating AMPs from the hemolymph of *C. sapidus* and *H. americanus* (Supplementary Data S1). Extraction methods were evaluated based on the total number of unique AMPs identified using two separate databases (AMPSphere for *C. sapidus* and the American lobster *H. americanus* proteome-derived AMP database), as shown in Figure 2A,B. Peptide sequences are reported using PEAKS Xpro notation, in which the periods “.” indicate the cleavage sites and the amino acids outside these markers represent the flanking amino acid residues from the parent protein sequence. For *C. sapidus*, Method 3 showed the highest extraction efficiency, identifying a mean of 32 AMPs across three biological replicates. This yield was significantly higher than that of all other tested methods, including the second most effective, Method 4 (*p* < 0.05). Method 4 also identified significantly more AMPs than Method 5 (*p* < 0.05). Methods 1 (12 AMPs), 2 (11 AMPs), and 5 (13 AMPs) produced the lowest number of unique AMPs, with no significant differences observed among them. For *H. americanus*, AMP candidates were identified from genome-derived AMP protein precursors rather than curated, experimentally validated AMP databases. To strengthen confidence in these identifications, candidate AMPs were evaluated using both DBAASP and AMPScanner, and in silico predicted AMPs were defined as those sequences supported by both models. A similar trend was observed in *H. americanus*: Method 3 was again the most efficient, identifying an average of 85 AMPs, which was significantly higher than the yields obtained with Method 2 (*p* < 0.01) and Method 5 (*p* < 0.01). Interestingly, Method 1 followed with 65 AMPs, and Method 4 identified 61 AMPs. Although both Method 1 and Method 4 were significantly more effective than the other methods (Method 1 vs 2, *p* < 0.05; Method 4 vs 5, *p* < 0.05), there was no statistically significant difference between Method 1 and Method 4. Consistent with the *C. sapidus* results, Methods 2 (23 AMPs) and 5 (27 AMPs) produced the lowest extraction yields in *H. americanus*. It is important to note that the physicochemical profile of the extracted peptides varied depending on the extraction method. Specifically, Methods 3 and 4 yielded a substantially broader range of peptide hydrophobicity, as indicated by the distribution of Grand Average of Hydropathy (GRAVY) scores, compared with the narrower hydrophobicity ranges observed in the other methods (Figure 2C). Distinct species-specific differences were also observed: AMPs from *C. sapidus* generally exhibited higher (more hydrophobic) GRAVY scores across most methods, whereas those from *H. americanus* had a trend toward neutral or hydrophilic GRAVY scores.

**Figure 2:**
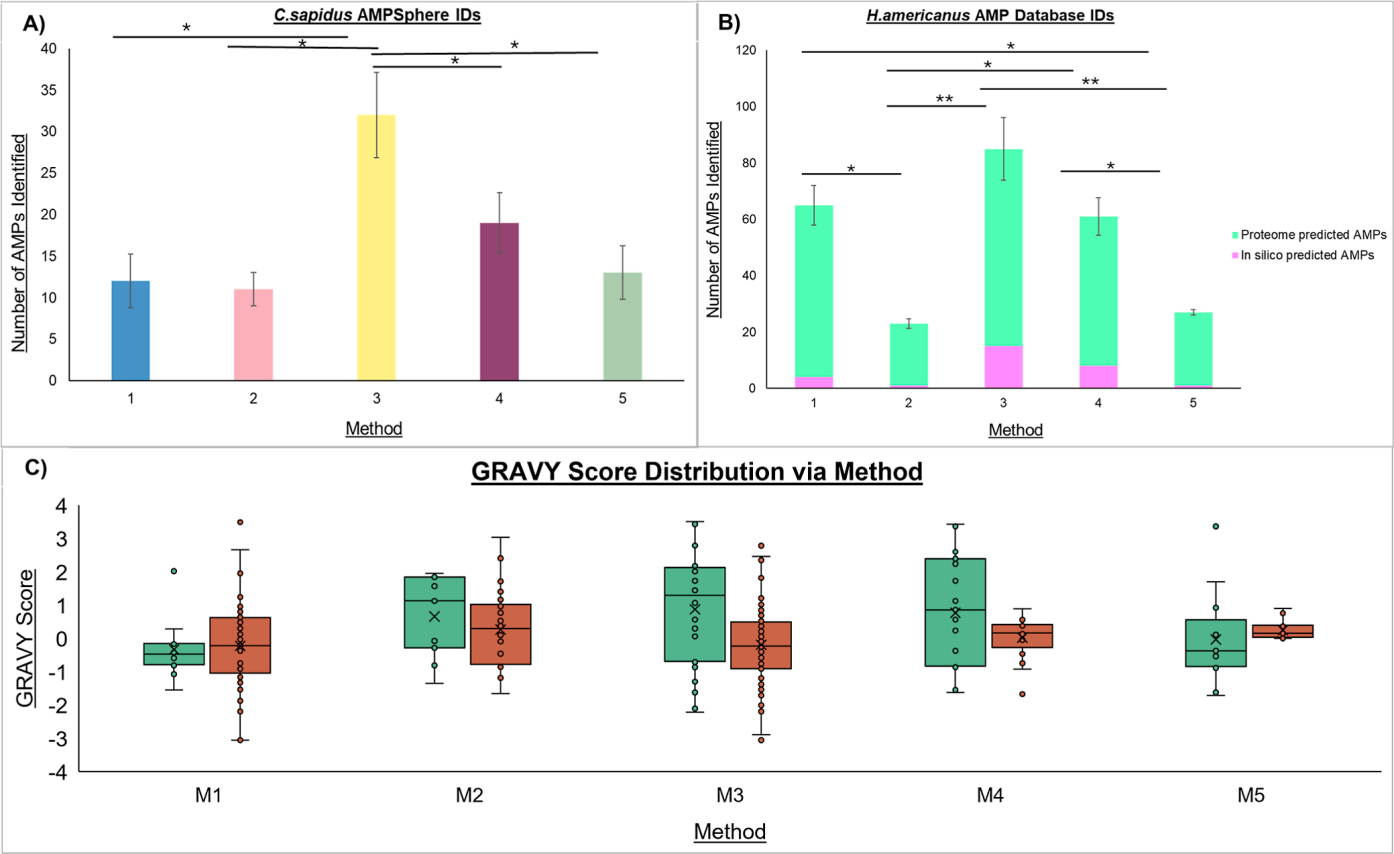
Identification of antimicrobial peptides (AMPs) from the hemolymph of two crustacean species, *C. sapidus* and *H. americanus*. (A) The number of unique AMP sequences identified across five different extraction methods (M1–M5) in *C. sapidus* from the AMPSphere database. (B) The number of unique AMP sequences identified across five different extraction methods (M1–M5) in *H. americanus*’s genome-derived AMPs database. (C) Comparison of the Grand Average of Hydropathicity (GRAVY) Score by species and extraction method. The horizontal line within each box represents the median (50th percentile), and the box boundaries represent the first (Q1) and third (Q3) quartiles. The overlaid dots indicate the GRAVY score of each individual peptide sequence. Statistical analysis comparing the means of IDs between methods was performed using a one-way ANOVA, followed by Tukey’s Honestly Significant Difference (HSD) post hoc test. Asterisks show statistically significant differences between methods. * *p*-value <0.05, ** *p*-value <0.001.

The distribution of *H. americanus* AMP families identified across all extraction methods is presented in Figure S1. The classifications were based on annotations from the proteome database, which enabled categorization into specific AMP families. The diversity of AMPs identified in *H. americanus* hemolymph highlights the complexity of the lobster’s innate immune system. The high prevalence of histone-derived AMPs is consistent with the established role of histones as latent immune effectors that are rapidly mobilized through proteolytic cleavage. Although traditionally associated with chromatin organization, histones (particularly H2A and H2B) are frequently processed to release N-terminal AMPs that function as immune defenses in marine invertebrates.^32–34^ This processing is often mediated by proteases such as cathepsins, enabling a synthesis-independent response to stress or infection.^35,36^ Histone-derived AMPs were consistently detected across extraction protocols, suggesting that these peptides are both abundant and resilient to variations in sample preparation. Antilipopolysaccharide factors (ALFs) also contributed substantially to the *H. americanus* AMP repertoire. ALFs are broad-spectrum AMPs characterized by a conserved disulfide-bonded lipopolysaccharide-binding domain that targets Gram-negative bacterial membranes common in marine environments.^37^ Functional diversification among ALF isoforms enables activities against bacteria, fungi, and certain enveloped viruses.^38,39^ Crustins, another prominent family, further enhances the immune landscape. These cysteine-rich peptides contain a C-terminal Whey Acidic Protein (WAP) domain associated with both antimicrobial activity and protease inhibition.^40^ In *H. americanus*, Crustins likely function in direct antibacterial defense against Gram-positive bacteria while also limiting host tissue damage during immune activation.^41^ Collectively, the co-occurrence of histone-derived AMPs, ALFs, and Crustins across extraction methods supports the conclusion that the evaluated workflows provide broad and complementary coverage of the *H. americanus* AMP repertoire. Although individual methods may preferentially enrich specific peptide classes, the observed diversity underscores both the robustness of the extraction approaches and the multifaceted nature of crustacean innate immunity, which relies exclusively on innate defenses to respond to microbial challenge and environmental stress.^42^

#### Impact of Extraction Modifications on AMP Yield

The results show that Method 3 is the most effective protocol for high-yield isolation of AMPs from crustacean hemolymph, particularly in *C. sapidus*. Method 3 is distinguished by three key modifications relative to the simplest protocol (Method 1): (1) pretreatment of hemolymph with an anticoagulant buffer (AB) and protease inhibitor (PI), (2) separation through a 10 kDa MWCO filter, and (3) omission of the DTT/IAA reduction and alkylation step. The substantial improvement in AMP yield observed for both Method 3 and Method 4 strongly suggests that the initial pretreatment with AB and PI, followed by MWCO filtration are critical factors. The AB/PI pretreatment minimizes peptide loss due to clotting and degradation by endogenous proteases. The EDTA and citrate in the AB prevent hemocyte aggregation and coagulation, which can physically trap small peptides, while the PI and low pH AcMeOH extraction suppress enzymatic degradation.^15^ The MWCO filter then effectively separates lower molecular weight AMPs from larger background proteins (>10 kDa), simplifying the resulting mass spectra.

#### Effect of Reduction and Alkylation on AMP Identification

The comparison between Method 3 (no DTT/IAA) and Method 4 (with DTT/IAA) is key to understanding the impact of reduction and alkylation on AMP identification. In *C. sapidus*, the addition of reduction and alkylation in Method 4 resulted in a statistically significant ∼40% decrease in unique AMP identifications compared with Method 3 (*p* < 0.001). Although reduction and alkylation are standard procedures for linearizing disulfide-bonded peptides to facilitate sequencing,^21^ many AMPs, such as defensins, require intact disulfide bonds to maintain their characteristic compact structure and biological activity.^43^ The data suggest that the DTT/IAA treatment introduces peptide loss due to additional handling steps and prolonged incubation (e.g., 30 min at 37 °C for DTT). Specifically, these losses are likely attributed to increased surface absorption of peptides during extended incubation, as well as potential ion suppression from residual reagents, which can disproportionately affect the detection of low-abundance AMPs. These findings indicate that omitting reduction and alkylation (Method 3) is advantageous for overall AMP identification, whereas Method 4 is more appropriate for specifically profiling defensins or other disulfide-rich peptides. Methods 2 and 5 consistently produced the lowest number of identified AMPs. Method 5, which selectively extracted peptides only from the hemocyte pellet after discarding the hemolymph supernatant, suggests that most AMPs in both *C. sapidus* and *H. americanus* are present in the hemolymph fraction rather than retained in hemocytes (Figure S2). This distribution indicates that these AMPs likely function as constitutive components of the innate immune response or are rapidly released from the hemocyte granules into the hemolymph upon withdrawal.

#### AMP Predictions

A total of 9 putative novel AMPs were predicted in *C. sapidus* and 11 in *H. americanus* (Table S1). In both species, the putative novel AMPs were characterized by overlapping, truncated peptide patterns. For example, in *H. americanus*, multiple truncated variants shared conserved motifs such as FWGMLK and FWGRLAK, whereas *C. sapidus* exhibited similar patterns, including AVNRLLYR and related sequences. These truncated peptides may originate from proteolysis or sample processing related degradation. However, their consistently high Total Local Confidence (TLC ≥ 85%) and reproducibility across biological replicates suggest that they may not be entirely stochastic. Truncated peptides are frequently reported as functionally relevant in antimicrobial peptide systems,^44^ where shorter sequences can retain essential cationic or amphipathic features required for activity^45^ and may even exhibit equal or greater antimicrobial potency compared with full-length precursors.^46^ Accordingly, the truncated variants identified here may represent biologically processed, functional forms rather than solely degradation artifacts. Nevertheless, the data presented here are based on MS identification and *in silico* prediction and therefore remain putative. Interestingly, the peptide AVLLPKKTEKK was identified across all methods in *H. americanus*. This peptide was predicted to be an AMP by DBAASP and CAMPR3 but not by AMPScanner; therefore, it was not classified as a putative AMP in this study. However, this same peptide has been previously reported in *C. sapidus* in response to low pH stress.^47^ This observation supports growing evidence that AMPs act not only as pathogen-neutralizing molecules but also as signaling mediators during environmental stress responses.^48,49^ Future studies should focus on quantitative profiling of truncated versus full-length potential AMPs, followed by peptide synthesis and biological functional validation.

### Neuropeptides

#### Recovery of Neuropeptides in the American Lobster Hemolymph

Five extraction methods were evaluated for their efficiency in isolating circulating neuropeptides from *C. sapidus* and *H. americanus* hemolymph. Lobster hemolymph is known to undergo rapid coagulation, often within 1 min of collection, due to activation of the prophenoloxidase cascade and associated proteolytic processes.^50^ Accordingly, our analysis focused on mature neuropeptides, defined as fully processed peptides generated from precursor prohormones through canonical cleavage at mono- or dibasic residues and associated PTMs. Because a genome-derived proteome database is available for *H. americanus*, restricting analysis to full-length mature peptides enabled confident evaluation of extraction efficiency while avoiding ambiguity arising from partially processed or degradation products.

The number and diversity of predicted mature neuropeptides recovered from *H. americanus* hemolymph varied substantially across extraction methods (Table 1, Figure S3(1–6)). Method 1, which relied solely on acidified methanol extraction, recovered only a single orcokinin peptide. In contrast, inclusion of a 10 kDa MWCO filtration step in Method 2 enabled detection of two additional orcokinins, likely by reducing ion suppression through removal of high-abundance hemolymph proteins. The most comprehensive neuropeptide recovery was achieved with Method 3, which incorporated anticoagulants and protease inhibitors prior to acidified methanol extraction, followed by MWCO filtration. This workflow enabled the detection of five orcokinin peptides and the myosuppressin pQDLDHVFLRFa, indicating superior preservation of peptide integrity and diversity. Orcokinin and myosuppressin neuropeptides are among the most abundant and widely distributed neuropeptides in American lobster neural and endocrine tissues,^51,52^ supporting their physiological relevance as circulating signaling molecules. Given the rapid coagulation and pronounced proteolytic activity in lobster hemolymph, early addition of anticoagulants and protease inhibitors likely stabilized labile neuropeptides prior to extraction. Method 4, which added a reduction and alkylation step conducted at room temperature or higher near the end of the workflow, yielded fewer peptides than Method 3, suggesting that additional handling steps may introduce peptide loss without offering substantial benefit for neuropeptide analysis. Finally, Method 5, which targeted endogenous peptides within isolated hemocytes, failed to recover any neuropeptides, supporting the premise that neuropeptides function as circulating hormones present in the hemolymph plasma rather than intracellular storage products.

**Table 1:** List of Predicted Mature Neuropeptides Detected in the American Lobster Hemolymph^a^

family	peptide	mass	method 1	method 2	method 3	method 4	method 5
Orcokinin	NFDEIDRSGFGFH	1539.67	x	x	x	x	
	NFDEIDRSGFGFN	1539.67		x	x	x	
	NFDEIDRSGFGFV	1501.68		x	x	x	
	FDAFTTGFGH	1212.52			x	x	
	VYGPRDIANLY	1279.66			x		
Myosuppressin	pQDLDHVFLRFa	1270.65			x		

a amidated; p: pyro-glutamination; x: detected using the specified extraction method.

#### Recovery of Neuropeptides in the Blue Crab Hemolymph

Unlike *H. americanus*, whose well-annotated genome enables confident identification of mature neuropeptides, neuropeptide analysis in *C. sapidus* remains more dependent on existing empirical neuropeptide databases and known crustacean neuropeptide motifs. Therefore, the data reported here pertain to previously reported neuropeptides in the crustacean nervous system or peptides matching established neuropeptide family motifs. Many of these predicted neuropeptides have been shown to modulate stomatogastric activity and stress responses in crustaceans through electrophysiological assays and quantitative peptidomics, underscoring the reliability of our MS-based workflow for capturing biologically significant neuropeptides from the hemolymph.^47,53,54^ Neuropeptide recovery from *C. sapidus* hemolymph varied substantially across extraction methods, with no single workflow providing comprehensive coverage of all detected peptide families (Table 2, Figure S4(1–8)). This heterogeneity reflects the sensitivity of the blue crab hemolymph matrix to differences in sample handling.

**Table 2:** List of Predicted Mature Neuropeptides Detected in the Blue Crab Hemolymph^a^

family	peptide	mass	method 1	method 2	method 3	method 4	method 5
AST-B	VPNDWAHFRGSWa	1469.70		x			x
	WLQLVCLWa	1058.57			x		
Myosuppressin	pQDLDHVFLRFa	1270.65		x	x	x	
Tachykinin	APSGFLGM(O)Ra	949.48	x		x		x
	APSGFLGMRa	933.49			x		
RYamide	SGFYANRYa	975.46					x
	EWYSQRYa	1029.47					x
YRamide	HIGSLYRa	843.47		x			x

a Amidated; (O): oxidation; p: pyro-glutamination; x: detected using the specified extraction method; AST-B: B-type allatostatin.

Method 1, employing only acidified methanol extraction, exhibited limited efficacy and recovered only a single tachykinin peptide. Incorporation of MWCO filtration in Method 2 improved recovery, enabling detection of peptides from three families (AST-B, myosuppressin, and YRamide). On the other hand, Methods 3 and 4, which introduced anticoagulants and protease inhibitors prior to extraction, recovered a broader but largely overlapping set of predicted neuropeptides. Notably, Method 4 did not outperform Method 3, suggesting that reduction and alkylation steps provide limited benefit and may introduce unnecessary peptide loss in hemolymph neuropeptidomics. Unexpectedly, Method 5 yielded the broadest neuropeptide profile within the hemocyte fraction, including an AST-B, a tachykinin, two RYamides, and a YRamide. Because hemocytes are not traditionally regarded as sites of neuropeptide synthesis, these observations may reflect incomplete plasma-cell separation or nonspecific adsorption of neuropeptides to the hemocyte pellet during low-speed centrifugation. However, emerging evidence of neuropeptide-encoding transcripts in crayfish and shrimp hemocytes, raises the possibility that hemocytes may possess previously unrecognized neuropeptide expression.^55,56^ Further studies will be required to determine whether these peptides genuinely originate from crustacean hemocytes or represent methodological carryover from plasma. Overall, no single extraction strategy was sufficient to capture the full neuropeptidome of blue crab hemolymph, indicating that a multimethod approach (e.g., combining Methods 2 and 3) may be necessary for comprehensive neuropeptide profiling in *C. sapidus*.

#### Novel Putative Neuropeptides in Blue Crab Identified by *De Novo* Sequencing

In contrast to *H. americanus*, which benefits from a high-quality genome assembly released in 2021 ^57^ and the resulting genome-informed proteome database that enables confident neuropeptide identification,^51,52^ the *C. sapidus* genome remains poorly assembled and minimally annotated.^58^ Consequently, most peptide identifications in this species still rely on matching MS/MS spectra to previously reported crustacean neuropeptides rather than to a comprehensive species-specific database. To expand beyond known entries, we also searched for putative neuropeptides in *C. sapidus* hemolymph that are absent from existing databases. *De novo* sequencing using PEAKS generated candidate peptide sequences for all MS/MS spectra, and only those with a Total Local Confidence (TLC) score greater than 85% were retained. These high-confidence sequences were subsequently screened for hallmark motifs associated with major crustacean neuropeptide families. For each shortlisted candidate, manual inspection of the corresponding MS/MS spectrum was performed to verify continuous backbone cleavage and ensure accurate sequence interpretation.

Through this process, we identified six additional putative neuropeptides that met all criteria and displayed sequence characteristics consistent with B-type allatostatin and RYamide peptides. Their sequences, Average Local Confidence (ALC) values, masses, and the extraction methods in which they were detected are summarized in Table S2 with their corresponding MS/MS spectra shown in Figure S5(1–6). Although Method 1 yielded only a single neuropeptide identifiable through database searching, it accounted for the majority of novel putative neuropeptides uncovered through *de novo* sequencing. Methods 3 and 4 also recovered additional RYamide-like putative neuropeptides that were absent from existing databases. None of the newly identified putative neuropeptides have previously been reported in any crustacean species, and their functional roles remain unknown. However, their detection in the hemolymph of the blue crab suggests that these peptides may act as circulating hormones involved in the regulation of physiological processes.

## Concluding Remarks

In this study, we systematically compared five extraction workflows to evaluate their effectiveness in recovering endogenous peptides from the hemolymph of *H. americanus* and *C. sapidus*. Our results demonstrate that a general profiling workflow—incorporating early hemolymph stabilization with anticoagulants and protease inhibitors, followed by organic solvent extraction and selective depletion of high-abundance proteins—provides the most comprehensive coverage and the broadest hydrophobicity range of both AMPs and neuropeptides in these species. Although reduction and alkylation were evaluated as potential workflow components, the limited recovery of disulfide-bonded peptides suggests that further optimization—such as adjusting the concentrations of reducing and alkylating agents, reaction temperatures, and incubation times—is necessary to improve the detection of structurally complex peptides. Together, these findings support a practical dual-strategy recommendation for future biological and peptidomic investigations, in which a general profiling approach is used for broad peptide discovery and system-level characterization, complemented by a targeted approach when enrichment of cysteine-rich peptide classes is required. We also identified six previously uncharacterized putative neuropeptides in *C. sapidus* and presented the first *in silico*-guided characterization of the antimicrobial peptidomes of *C. sapidus* and *H. americanus*. In addition, peptide bioactivity in this study was inferred from conserved sequence motifs and computational predictions rather than validated experimentally. Future efforts integrating targeted functional assays, quantitative measurements, and physiological or immune-challenge experiments will be essential for defining the biological roles of these newly identified peptides and for evaluating their potential as antimicrobial therapeutics or neuromodulators. Overall, this work establishes a robust methodological framework for crustacean hemolymph peptidomics and provides a foundation for future discovery of novel AMPs and neuropeptide scaffolds in ecologically and economically important crustacean model systems. The extraction strategy insights and dual-workflow framework described here are also broadly applicable to other hemolymph-based systems, including insects and other arthropods, as well as additional invertebrate biofluid models, where rapid coagulation and high protease activity present similar challenges for stabilizing and profiling endogenous bioactive peptides.

## Supplementary Material





## Data Availability

The mass spectrometry proteomics data have been deposited to the ProteomeXchange Consortium via the MassIVE partner repository with data set identifier **MSV000100293**.

## Notes

Table S1: Putative novel AMPs from *C. sapidus* and *H. americanus* identified by *de novo* sequencing; Table S2: Putative neuropeptides detected in the hemolymph of the blue crab *C. sapidus* by *de novo* sequencing; Figure S1: Distribution of AMP families identified using the *H. americanus* proteome AMP database across all five methods; Figure S2: Venn diagram showing the distribution of AMPs identified in the hemolymph and hemocyte fractions of *C. sapidus* and *H. americanus*; Figure S3.1–6: MS/MS spectra from PEAKS software for neuropeptides detected in the American lobster *H. americanus* hemolymph; Figure S4.1–8: MS/MS spectra from PEAKS software for neuropeptides detected in the blue crab *C. sapidus* hemolymph; Figure S5.1–6: MS/MS spectra from PEAKS software for putative neuropeptides detected in the blue crab *C. sapidus* hemolymph (PDF)
Supplementary Data S1: Putative AMPs identification results exported from PEAKS Studio Xpro software (XLSX)
