# Supplementary material for: Mass Spectrometry-Based Peptidomics for the Discovery and Profiling of Endogenous Peptides in Crustacean Hemolymph
Source: ACS Omega. 2026 May 5;11(19):28529–38. doi: 10.1021/acsomega.6c00679 (PMC13191662; doi:10.1021/acsomega.6c00679)
Supplement: Supplementary file 1 [file ao6c00679_si_001.pdf]

Supporting Information

Mass Spectrometry-Based Peptidomics for the Discovery and Profiling of Endogenous Peptides in Crustacean Hemolymph

Tina C. Dang[1, †], Vu Ngoc Huong Tran[1, †], Satirtha Saha Protya[1], Margot Beaver[1], King Wai Yi[2], Lingjun Li[1,3,4,5,*]

[1]School of Pharmacy, University of Wisconsin-Madison, Madison, WI 53705

[2]Madison West High School, 30 Ash Street, Madison, WI 53726

[3]Department of Chemistry, University of Wisconsin-Madison, Madison, WI 53706

[4]Lachman Institute for Pharmaceutical Development, School of Pharmacy, University of Wisconsin-Madison, Madison, WI 53705, United States

[5]Wisconsin Center for NanoBioSystems, School of Pharmacy, University of Wisconsin-Madison, Madison, WI 53705, United States

[†]Equal contribution

*Corresponding author

[†]Equal contribution

Tel.: +1 (608) 265-8491

Fax: +1 (608) 262-5345

E-mail: lingun.li@wisc.edu

Table of Contents

Table S1. Putative novel AMPs from *Callinectes sapidus* and *Homarus americanus* identified by *de novo* sequencing.

Table S2. Putative neuropeptides detected in the hemolymph of the blue crab *Callinectes sapidus* by *de novo* sequencing.

Figure S1. Distribution of AMP families identified using the *H. americanus* proteome AMP database across all five methods.

Figure S2. Venn diagram showing the distribution of AMPs identified in the hemolymph and hemocyte fractions of *Callinectes sapidus* and *Homarus americanus.*

Figure S3.1-6. MS/MS spectra from PEAKS software for neuropeptides detected in the American lobster *Homarus americanus* hemolymph.

Figure S4.1-8. MS/MS spectra from PEAKS software for neuropeptides detected in the blue crab *Callinectes sapidus* hemolymph.

Figure S5.1-6. MS/MS spectra from PEAKS software for putative neuropeptides detected in the blue crab *Callinectes sapidus* hemolymph.

Table S1. Putative novel AMPs from *Callinectes sapidus* and *Homarus americanus* identified by *de novo* sequencing. A sequence that has a probability greater than 0.5 is considered as AMP per AMPScanner v.2.'s manual. All sequences were predicted to be AMP by two independent AMP prediction algorithms developed by AMPScanner and DBAASP.

Sequence	Species	DDBAASP Prediction	AMPScanner Prediction	CAMPR3 Prediction	MotifQuest Motif	MotifQ Sco
LVRSLPS	*C. sapidus*	AMP	0.5795	AMP	LVRSLPS	53.96
KVGFKLYPNR	*C. sapidus*	AMP	0.8514	AMP	KVGFKLYPNR	64.80
EKAVNRLLYR	*C. sapidus*	AMP	0.8386	AMP	RLLYR	34.98
QQAVNRLLYR	*C. sapidus*	AMP	0.5416	AMP	RLLYR	34.98
AVNRLLYR	*C. sapidus*	AMP	0.8466	AMP	RLLYR	34.98
QAVNRLLYR	*C. sapidus*	AMP	0.6945	AMP	RLLYR	34.98
VNRLLYR	*C. sapidus*	AMP	0.8316	AMP	RLLYR	34.98
RLLYRLY	*C. sapidus*	AMP	0.7541	AMP	RLLYR	34.98
RLLYR	*C. sapidus*	AMP	0.598	AMP	RLLYR	34.98
FWGMLK	*H. americanus*	AMP	0.9117	AMP	FWGMLK	19.94
FWGMLKKL	*H. americanus*	AMP	0.9988	AMP	FWGMLK	19.94
FWGMLKKLLF	*H. americanus*	AMP	0.9979	AMP	FWGMLK	19.94
FWGMLKKLL	*H. americanus*	AMP	0.9994	AMP	FWGMLK	19.94
FWGMLKK	*H. americanus*	AMP	0.9924	AMP	FWGMLK	19.94
FWGRLAKGVLSSFFE	*H. americanus*	AMP	0.9944	AMP	FWGMLK	19.94
FWGRLAKGVLSSF	*H. americanus*	AMP	0.9999	AMP	FWGMLK	19.94
FWGRLAKGVLSS	*H. americanus*	AMP	0.9986	AMP	FWGMLK	19.94
FWGRLAKGVLS	*H. americanus*	AMP	0.9998	AMP	FWGMLK	19.94
FWGRLAKGVL	*H. americanus*	AMP	0.9998	AMP	FWGMLK	19.94
FWGRLAKGVLT	*H. americanus*	AMP	0.9994	AMP	FWGMLK	19.94

Table S2. Putative neuropeptides detected in the hemolymph of the blue crab *Callinectes sapidus* by *de novo* sequencing.

Family	Peptide	ALC (%)	Mass	Method 1	Method 2	Method 3	Method 4	Method 5
AST-B	WLAHKGVWa	91	994.55	x				
	WLAHRGVWa	89	1022.56	x				
RYamide	WLKRYa	88	763.45	x				
	YNDVALVVQDRYa	85	1452.74	x				
	RFLPPAFQRYa	95	1292.71			x		
	ADLAHKQQAVNRSRYa	91	1754.93				x	

a: amidated; x: detected using the specified extraction method; ALC: Average Local Confidence; AST-B: B-type allatostatin

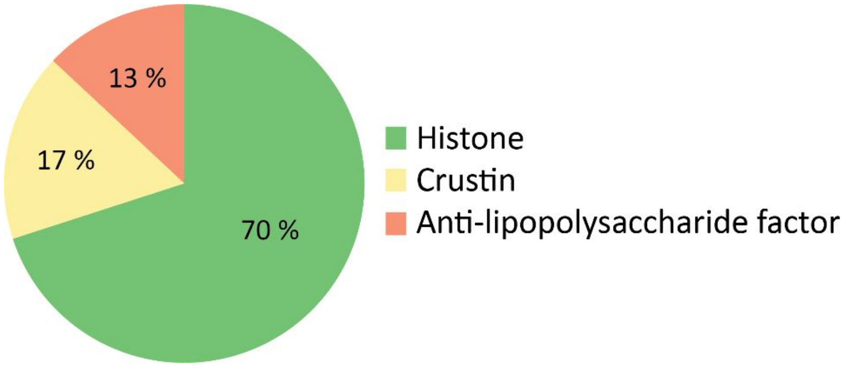

Figure S1. Composition of identified antimicrobial peptide (AMP) families in *Homarus americanus*. The pie chart illustrates the relative results of the three primary AMP families; Anti-lipopolysaccharide factors (ALFs), Crustins, and Histone-derived peptides detected across all extraction methods. Family assignments were determined based on existing annotations within the curated *H. americanus* proteome.

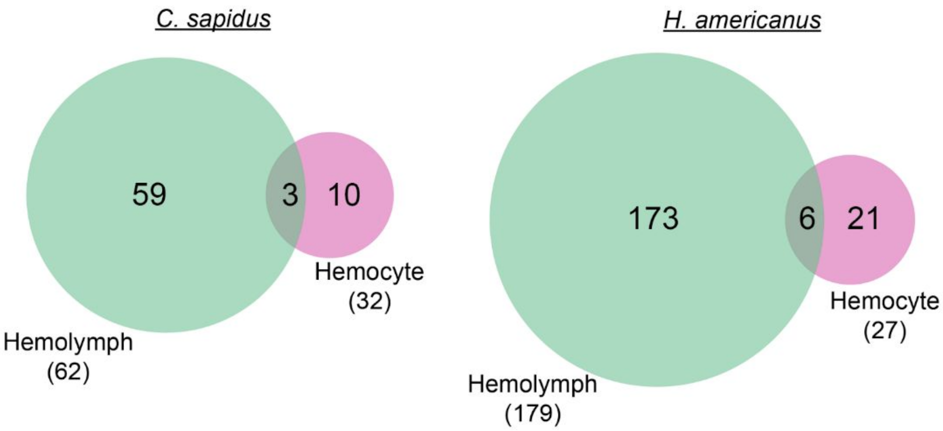

Figure S2. Distribution of AMPs in hemolymph and hemocyte fractions. Venn diagrams comparing the AMPs identified in *C. sapidus* and *H. americanus*. Method 5 (M5) was used to isolate hemocyte pellets after removing the hemolymph. Results indicate that most identified AMPs are localized in the hemolymph rather than sequestered in hemocytes.

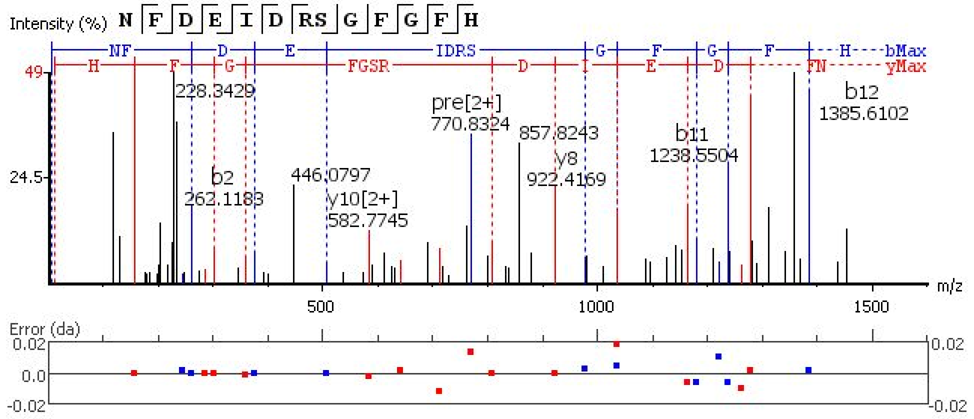

Figure S3.1. MS/MS spectrum from PEAKS software for an Orcokinin neuropeptide with the sequence NFDEIDRSGFGFH detected in *H. americanus* hemolymph.

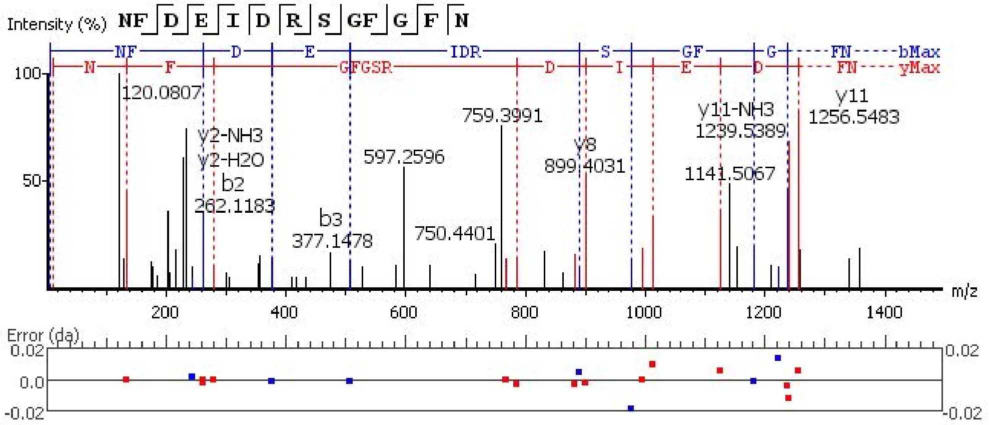

Figure S3.2. MS/MS spectrum from PEAKS software for an Orcokinin neuropeptide with the sequence NFDEIDRSGFGFN detected in *H. americanus* hemolymph.

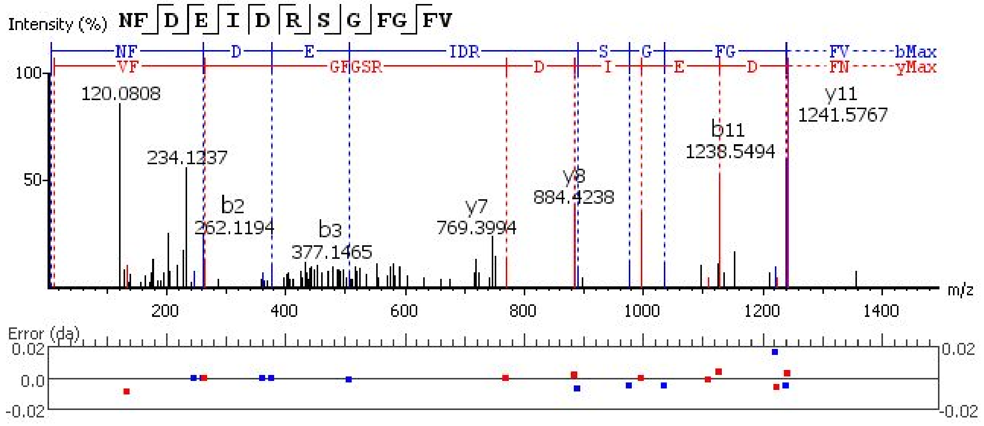

Figure S3.3. MS/MS spectrum from PEAKS software for an Orcokinin neuropeptide with the sequence NFDEIDRSGFGFV detected in *H. americanus* hemolymph.

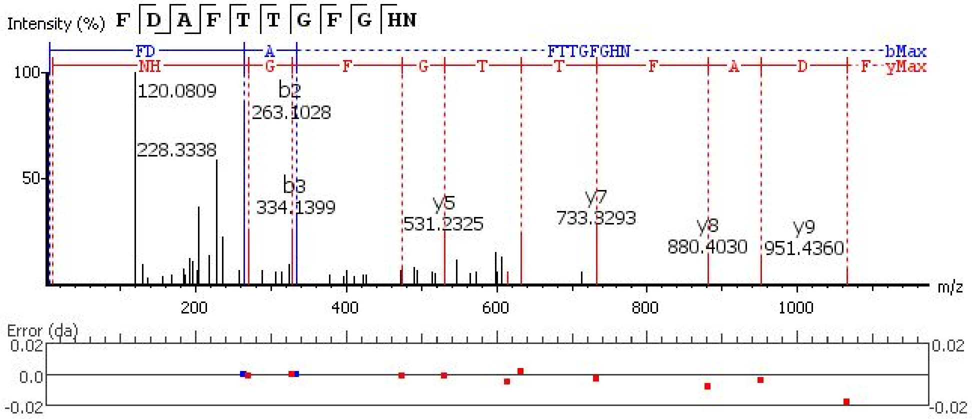

Figure S3.4. MS/MS spectrum from PEAKS software for an Orcokinin neuropeptide with the sequence FDAFTTGFGHN detected in *H. americanus* hemolymph.

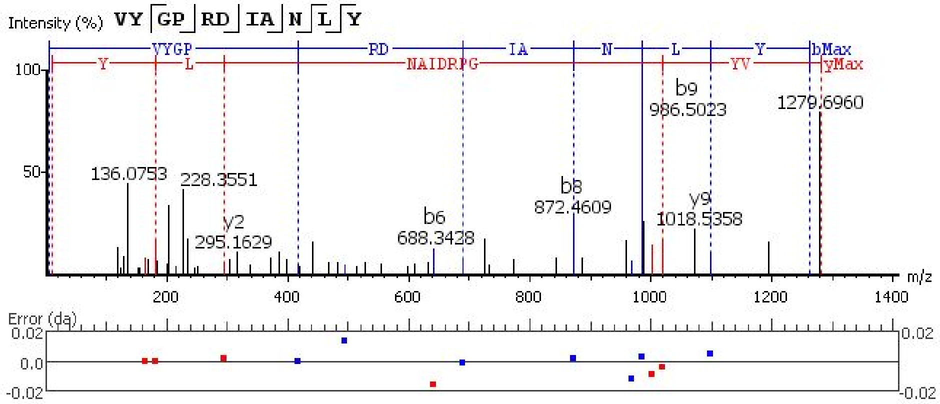

Figure S3.5. MS/MS spectrum from PEAKS software for an Orcokinin neuropeptide with the sequence VYGPRDIANLY detected in *H. americanus* hemolymph.

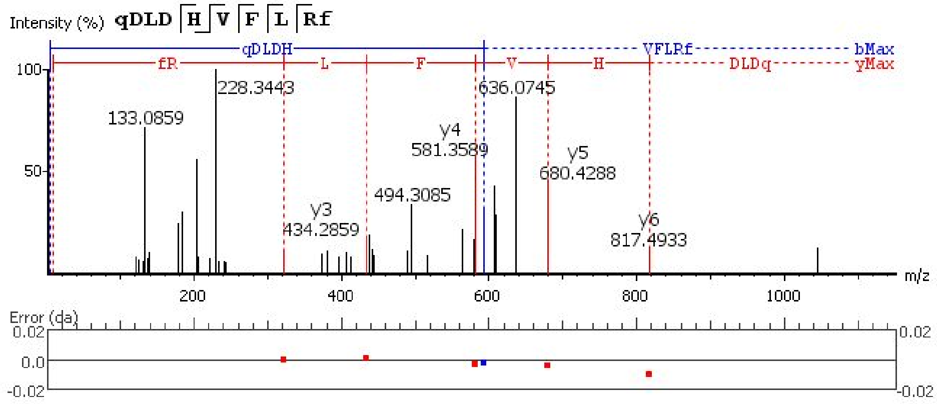

Figure S3.6. MS/MS spectrum from PEAKS software for an Orcokinin neuropeptide with the sequence pQDLDHVFLRFamide detected in *H. americanus* hemolymph.

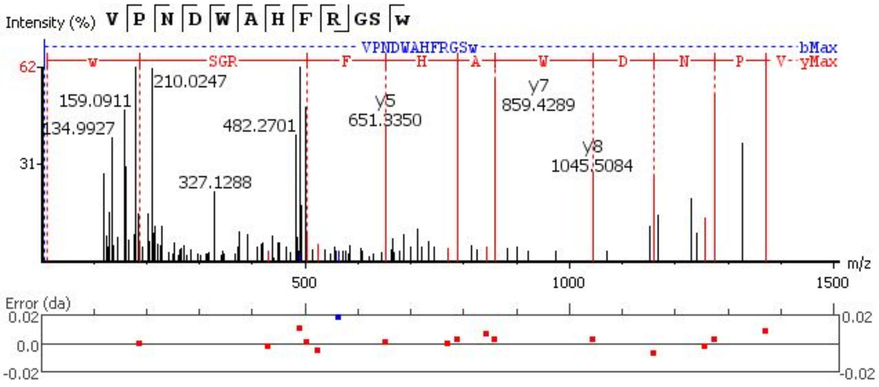

Figure S4.1. MS/MS spectrum from PEAKS software for an AST-B neuropeptide with the sequence VPNDWAHFRGSWamide detected in *C. sapidus* hemolymph.

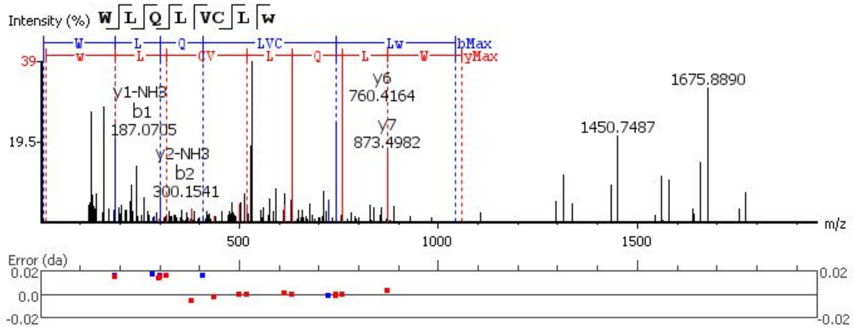

Figure S4.2. MS/MS spectrum from PEAKS software for an AST-B neuropeptide with the sequence WLQLVCLWamide detected in *C. sapidus* hemolymph.

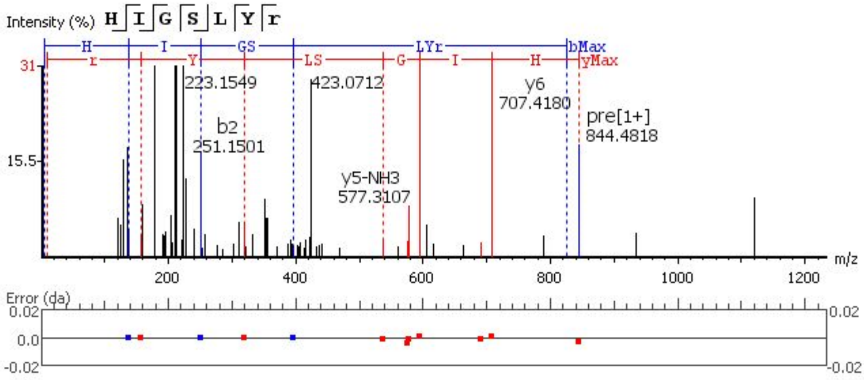

Figure S4.3. MS/MS spectrum from PEAKS software for a neuropeptide with the sequence HIGSLYRamide detected in *C. sapidus* hemolymph.

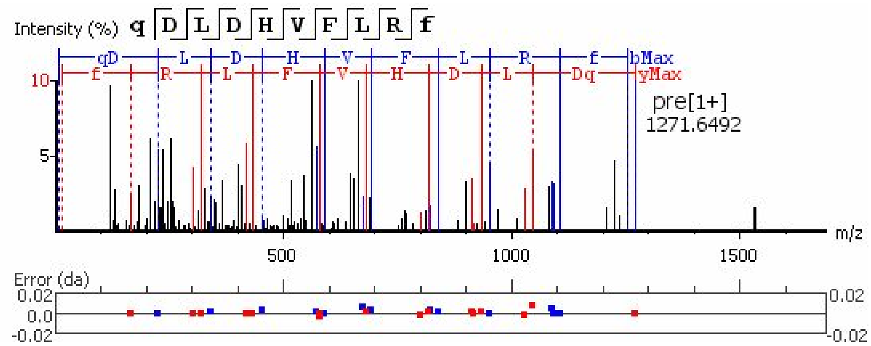

Figure S4.4. MS/MS spectrum from PEAKS software for a Myosuppressin neuropeptide with the sequence pQDLDHVFLRFamide detected in *C. sapidus* hemolymph.

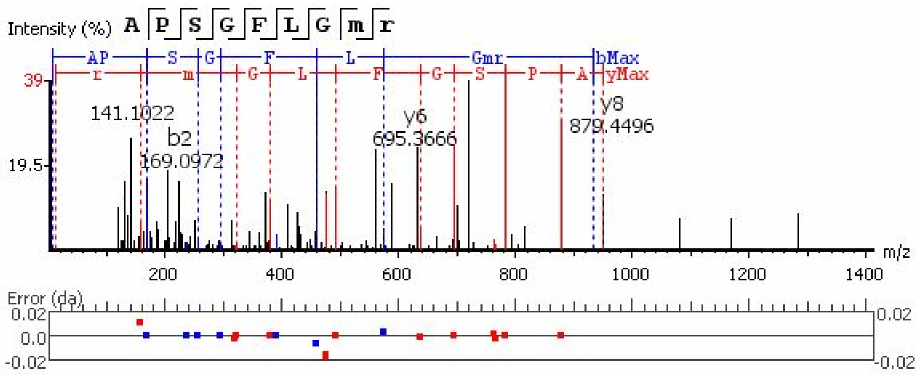

Figure S4.5. MS/MS spectrum from PEAKS software for a Tachykinin neuropeptide with the sequence APSGFLGM(O)Ramide detected in *C. sapidus* hemolymph.

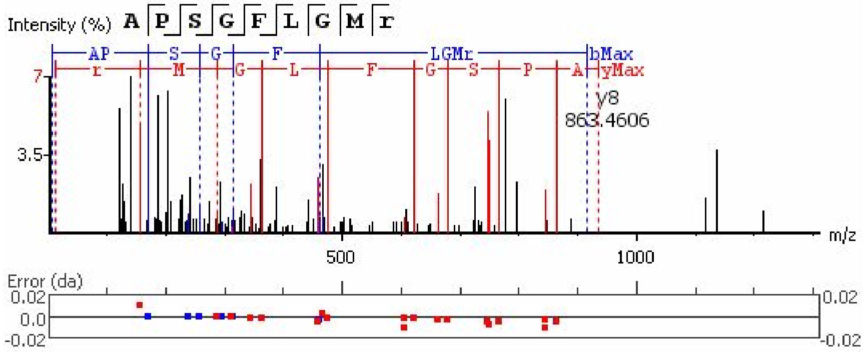

Figure S4.6. MS/MS spectrum from PEAKS software for a Tachykinin neuropeptide with the sequence APSGFLGMRamide detected in *C. sapidus* hemolymph.

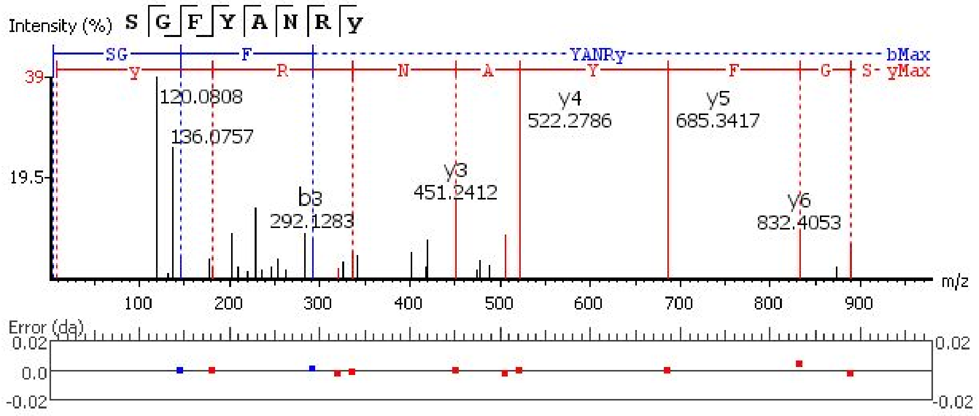

Figure S4.7. MS/MS spectrum from PEAKS software for a RYamide neuropeptide with the sequence SGFYANRYamide detected in *C. sapidus* hemolymph.

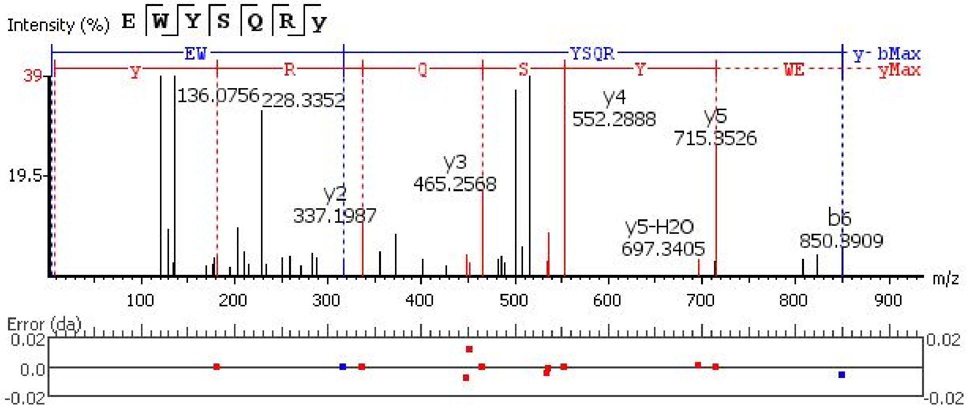

Figure S4.8. MS/MS spectrum from PEAKS software for a RYamide neuropeptide with the sequence EWYSQRYamide detected in *C. sapidus* hemolymph.

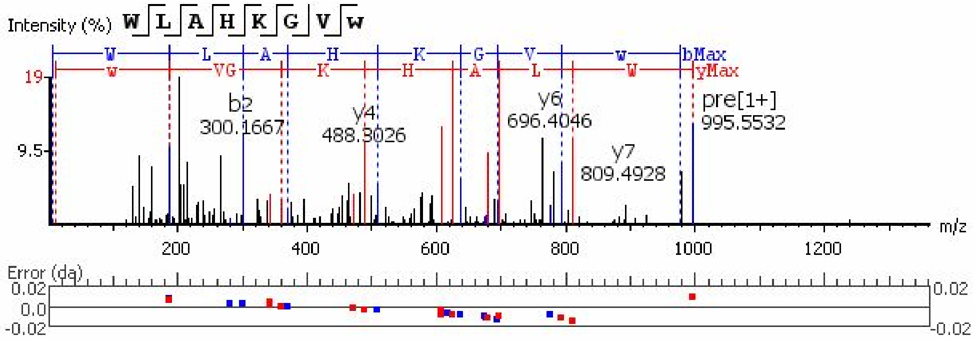

Figure S5.1. MS/MS spectra from PEAKS software for a putative AST-B neuropeptide with the sequence WLAHKGVWamide detected in the blue crab *Callinectes sapidus* hemolymph.

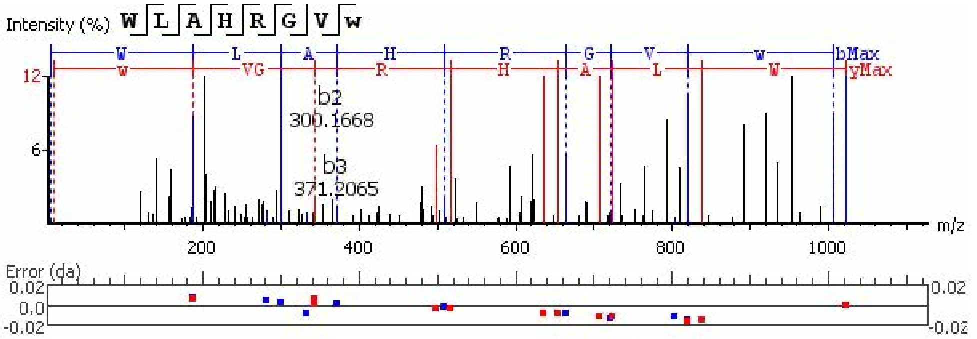

Figure S5.2. MS/MS spectra from PEAKS software for a putative AST-B neuropeptide with the sequence WLAHRGVWamide detected in the blue crab *Callinectes sapidus* hemolymph.

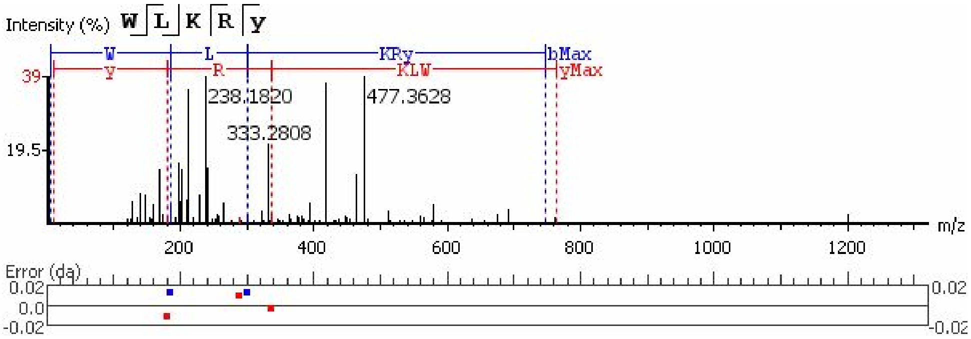

Figure S5.3. MS/MS spectra from PEAKS software for a putative RYamide neuropeptide with the sequence WLKRYamide detected in the blue crab *Callinectes sapidus* hemolymph.

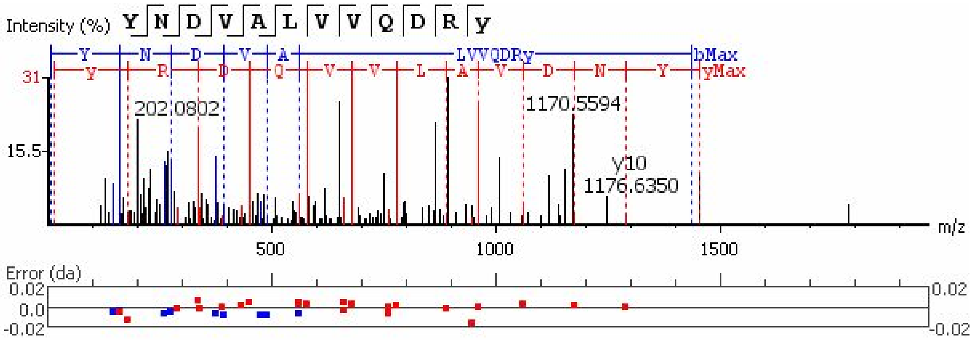

Figure S5.4. MS/MS spectra from PEAKS software for a putative RYamide neuropeptide with the sequence YNDVALVVQDRYamide detected in the blue crab *Callinectes sapidus* hemolymph.

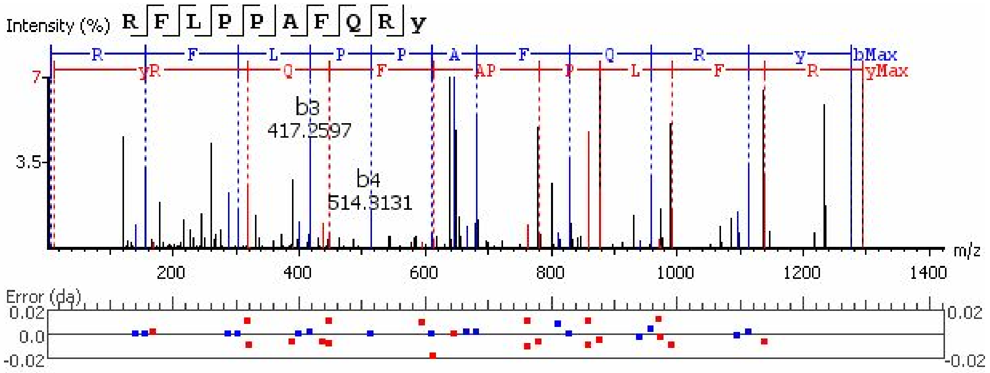

Figure S5.5. MS/MS spectra from PEAKS software for a putative RYamide neuropeptide with the sequence RFLPPAFQRYamide detected in the blue crab *Callinectes sapidus* hemolymph.

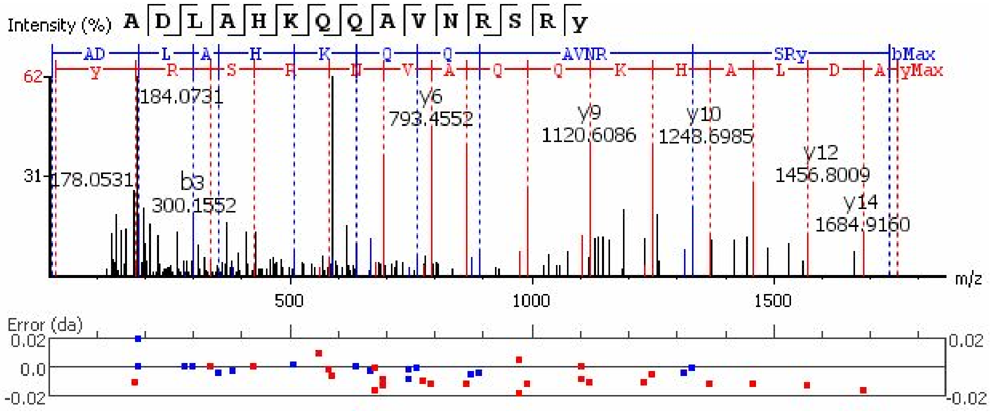

Figure S5.6. MS/MS spectra from PEAKS software for a putative RYamide neuropeptide with the sequence ADLAHKQQAVNRSRYamide detected in the blue crab *Callinectes sapidus* hemolymph.
